# Temperature–amplitude coupling for stable biological rhythms at different temperatures

**DOI:** 10.1371/journal.pcbi.1005501

**Published:** 2017-06-08

**Authors:** Gen Kurosawa, Atsuko Fujioka, Satoshi Koinuma, Atsushi Mochizuki, Yasufumi Shigeyoshi

**Affiliations:** 1 Theoretical Biology Laboratory, RIKEN, Wako, Japan; 2 Department of Anatomy and Neurobiology, Kindai University Faculty of Medicine, Osakasayama City, Osaka, Japan; King’s College London, UNITED KINGDOM

## Abstract

Most biological processes accelerate with temperature, for example cell division. In contrast, the circadian rhythm period is robust to temperature fluctuation, termed temperature compensation. Temperature compensation is peculiar because a system-level property (i.e., the circadian period) is stable under varying temperature while individual components of the system (i.e., biochemical reactions) are usually temperature-sensitive. To understand the mechanism for period stability, we measured the time series of circadian clock transcripts in cultured C6 glioma cells. The amplitudes of *Cry1* and *Dbp* circadian expression increased significantly with temperature. In contrast, other clock transcripts demonstrated no significant change in amplitude. To understand these experimental results, we analyzed mathematical models with different network topologies. It was found that the geometric mean amplitude of gene expression must increase to maintain a stable period with increasing temperatures and reaction speeds for all models studied. To investigate the generality of this temperature–amplitude coupling mechanism for period stability, we revisited data on the yeast metabolic cycle (YMC) period, which is also stable under temperature variation. We confirmed that the YMC amplitude increased at higher temperatures, suggesting temperature-amplitude coupling as a common mechanism shared by circadian and 4 h-metabolic rhythms.

## Author summary

Circadian rhythms govern the timing of many physiological events. Mysteriously, the period of the rhythm is robust to temperature although the underlying biochemical reactions usually accelerate with temperature, a paradox that has remained unsolved for more than 60 years. Experiments conducted over the last few decades in insects, mammals, and plants have demonstrated that biological rhythms are governed by cyclical changes in gene expression. However, the topologies of the regulatory network structures for these rhythms differ between species, suggesting that the mechanisms for period stability with temperature are not conserved. But is it true? We examined the mechanisms for period stability with temperature by combining computational models with distinct network structures and experimental observations from mammalian cell cultures. Unexpectedly, we found that temperature-sensitive amplitude of gene expression, which we call "temperature–amplitude coupling," can stabilize the period with temperature in all models studied, despite differences in regulatory network structures. Thus, the mechanisms for circadian period stability may be shared across species.

## Introduction

Many physiological processes are sensitive to temperature. At the biochemical level, the speed of the reactions tends to increase two- to three-fold with a 10°C temperature rise [1]. At the system level, cell growth accelerates with temperature, and the cell cycle period of NIH3T3 cells decreases to one-third as the temperature rises by 10°C [2]. In contrast, the circadian rhythm period is robust to temperature [1,3]. Notably, this property, so-called temperature compensation, is observed in both species without and with strong thermal homeostasis (poikilotherms and homeotherms). The definition of temperature compensation is that the oscillator period is constant at different but constant temperatures (0.85<*Q*_10_<1.15) [4]. For example, the circadian period of mammalian NIH3T3 cells remains roughly unchanged over a wide range of temperatures [2]. Similarly, the period of the yeast metabolic cycle (YMC), approximately 4 h under certain experimental conditions, is also robust to temperature [5].

How can the period of biological rhythms be stable under varying temperature [6–21]? Proposed hypothesis for this temperature compensation include the (i) "critical reaction hypothesis," (ii) "balance hypothesis," and (iii) "temperature–amplitude coupling hypothesis." The critical reaction hypothesis (i) posits that there are critical reactions governing the circadian period, and that the period can be stable if these critical reactions are insensitive to temperature [11]. Alternatively, the balance between period-lengthening and -shortening reactions (ii) could lead to a stable period [8,9]. Finally, temperature-sensitivity of circadian oscillation amplitude (iii) may stabilize the period [7]. The critical reaction hypothesis (i) is supported by experimental evidence from *cyanobacteria* [11] and mammals [14]. However, it still remains unclear how the circadian period can be maintained against temperature in which not only the critical reactions but also the other reactions in the network should affect the period. As for the temperature–amplitude coupling hypothesis (iii), there is yet no strong experimental evidence. For example, the amplitude of bioluminescence in the *Gonyaulax* circadian clock does decrease with temperature [6]. While the abovementioned hypothetical mechanisms for temperature compensation are not mutually exclusive, they may raise the question of whether any of abovementioned hypotheses is actually the mechanism of temperature compensation for circadian rhythms.

In *Drosophila*, *Neurospora*, mammals, and plants, diurnal activities are driven by transcriptional regulatory networks for genes and proteins [1]. Transcriptional regulatory network structures for circadian rhythms are similar between species in that there are both positive and also negative elements controlling gene-expression [22–24]. However, the topology of the regulatory network structures is not precisely the same across species, so the mechanisms of temperature compensation may also vary among species. Moreover, the mechanisms of temperature compensation for circadian rhythms are not necessarily the same as those for the yeast 4 h-metabolic cycle.

In this study, we examined the mechanisms for temperature compensation. First, we demonstrate temperature-sensitivity (or insensitivity) of the circadian clock system in C6 rat glioma cells. We then applied theoretical models to explain these results. Specifically, we addressed the question of whether period stability to temperature depends on the structures of regulatory networks by comparing experimental results to models with different structures. Finally, we revisited the time-series of the YMC [5] and discuss the possibility that the mechanisms for temperature compensation are shared between circadian and yeast metabolic rhythms.

## Results

### Stability of the circadian period and amplitude in the mammalian cultured cell

It has been reported that the expression time series of some genes in circadian clock system are sensitive to temperature whereas those of other genes are not [6,18]. To investigate the mechanisms for temperature compensation, we systematically examined the temperature sensitivities of clock gene expression levels in mammalian cultured cells.

**Fig 1:**
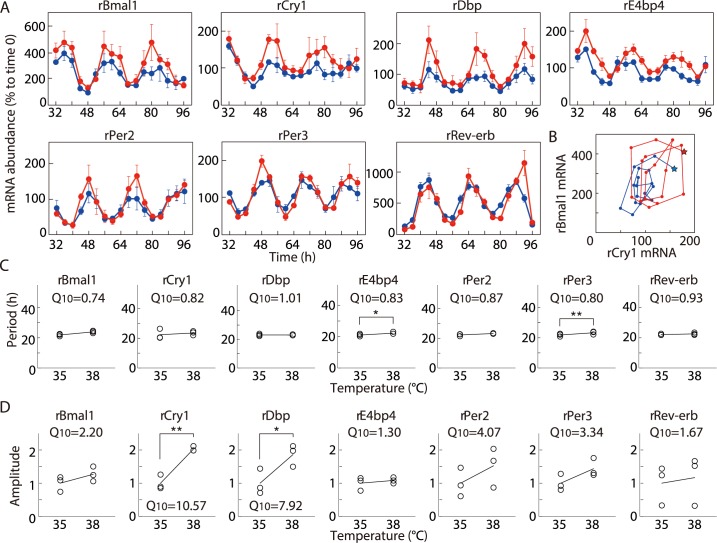
Temperature-dependent variation of circadian clock gene expression. (A) Representative time course of clock gene mRNA expression in C6 glioma cells at 35°C and 38°C. Results are expressed relative to time 0 (%) for clock mRNAs r*Bmal1*, r*Cry1*, r*Dbp*, r*E4bp4*, r*Per2*, r*Per3*, and r*Rev-erbα*. The values are means (±SEM) of at least three independent experiments. Blue circles/lines depict values obtained at 35°C and red circles those acquired at 38°C. (B) Oscillatory trajectory at 35°C (blue) and 38°C (red) plotted on a plane with x- and y-axes showing the amounts of r*Cry1* and r*Bmal1* mRNA, respectively. Stars represent the starting points of the trajectories. (C) Periods and *Q*_10_ values of the circadian rhythm at 35°C and 38°C. All data were obtained from three independent experiments (^*^p < 0.05 and ^**^p < 0.01 by Student’s t-test). Solid lines represent regression lines. (D) Amplitudes and *Q*_10_ values of the circadian rhythm at 35°C and 38°C. Values on the y-axis are fold changes over mean amplitudes for each gene at 35°C. The data were obtained from three independent experiments (^*^p < 0.05 and ^**^p < 0.01 by Student’s t-test). Solid lines represent regression lines.

We compared the expression time series of clock genes for C6 glioma circadian rhythms at 35°C and 38°C. In preliminary studies, the amplitude of the first cycle after the stimulation was markedly higher than those of following cycles. This finding suggests that, immediately after the stimulation, the state variable jumped out of the attractor of the circadian limit cycle and that the first cycle exhibited a relaxation process that did not trace the trajectory of the circadian limit cycle. Therefore, we excluded the first cycle and evaluated the mRNA expression levels of clock genes r*Bmal1*, r*Cry1*, r*Dbp*, r*E4bp4*, r*Per2*, r*Per3*, and r*Rev-erbα* mRNAs every 4 h from 32 to 96 h post-stimulation. During this period, the amplitude of the oscillation remained reasonably constant in the period after the temperature shift. (Fig 1A). Analyses of period and amplitude using ARSER [25] revealed that all genes examined showed a circadian rhythm with periods within the circadian range, from 21.0 to 23.6 h. We plotted the state points in chronological order with r*Cry1* and r*Bmal1* mRNA expression levels on the x- and y-axis, respectively (Fig 1B), and found that the oscillatory trajectory at 38°C was much larger than at 35°C.

To examine whether temperature affects the period length and amplitude, we calculated temperature coefficient (*Q*_10_) values, which expresses the reaction change over a 10°C increase in temperature of seven clock genes (Fig 1C and 1D). Although the *Q*_10_ values of the period varied between 1.01 (r*Dbp*) and 0.74 (r*Bmal1*), this range is deemed as temperature-compensated [4] and suggests that temperature compensation is preserved in C6 glioma cells. Amplitudes of both r*Cry1 (p* = 0.0068) and r*Dbp (p* = 0.043*)* mRNA expression levels increased significantly (Fig 1D) with, *Q*_10_ values of 10.57 and 7.92, respectively. All other genes also exhibited *Q*_10_ values of larger than unity. Because the *Q*_10_ values for biological systems are generally between 2 and 3 [4], oscillation amplitudes of most if not all are affected by temperature change.

### Temperature–amplitude coupling can stabilize oscillation period in a classical model of circadian rhythms

Do the observed temperature-dependent circadian expression levels of some genes (temperature–amplitude coupling) account for temperature compensation? To understand the experimental results, we analyzed a classical negative feedback model of circadian rhythms [26,27], the so-called Goodwin model [9,28,29], and investigated the conditions required for period stability. The regulatory networks of circadian rhythms are more complex than assumed by this model, so we compared the conditions for period stability between this simple model and a detailed model [30].

**Fig 2:**
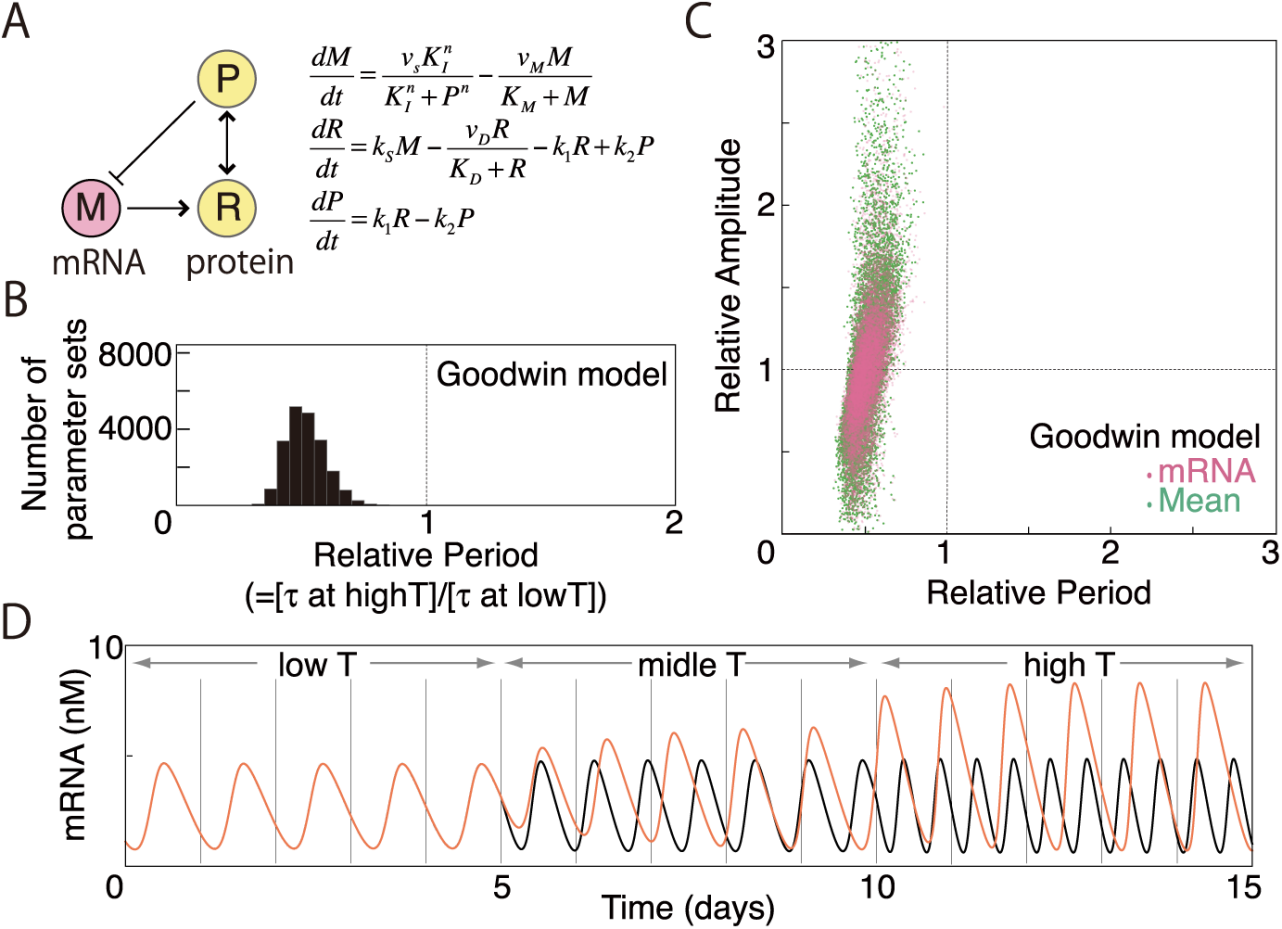
Random parameterization of a classical model to explain the gene expression data in Fig 1. (A) The classical model with negative feedback only (Goodwin model). Variables presented are mRNA concentration (*M*) and the concentration of a cytosolic protein (*R*) and nuclear protein (*P*) that downregulate the expression of this mRNA. (B) Distribution of period as reaction rates are increased. First, starting from the standard parameter set: *n* = 2, *v*_*s*_ = 1.6, *K*_*I*_ = 1, *v*_*m*_ = 0.505, *K*_*M*_ = 0.5, *k*_*s*_ = 0.5, *v*_*d*_ = 1.4, *K*_*D*_ = 0.13, *k*_*1*_ = 0.5, and *k*_*2*_ = 0.6, we prepared 500 basic parameter sets for which parameters *v*_*s*_, *v*_*m*_, *k*_*s*_, *v*_*d*_, *k*_*1*_, and *k*_*2*_ ranged from one-half and to double the standard values. Second, using the 500 basic parameter sets, we constructed 100 additional parameter sets for each basic parameter set in which *v*_*s*_, *v*_*m*_, *k*_*s*_, *v*_*d*_, *k*_*1*_, and *k*_*2*_ were increased randomly 1.5- to 2.5-fold above the basic parameter set values. The average parameter increase was ~2-fold. Of 50,000 parameter sets, sustained oscillations were obtained using 20,680. We plotted the ratio (relative period), defined as the new period with increased reaction speed divided by the basic period with basic parameter sets. (C) Distribution of both the oscillatory mRNA amplitude (*M*, pink) and the geometric mean (green) of *M*, *R*, and *P* relative amplitudes plotted as a function of relative period when reaction rates are increased (20,680 parameter sets). (D) Two example time series (black and red) of mRNA (*M*) at “low (20°C)”, “medium (25°C)”, and “high (30°C)” temperature. Parameter values at low temperature are the values of the standard parameter set above. Parameter values at different temperatures are changed according to the Arrhenius equation of $k_{i}=A_{i}e^{-E_{i}/RT}$, where *E*_*i*_ is activation energy. Activation energies (*E*_*i*_) of *v*_*s*_, *v*_*m*_, *k*_*s*_, *v*_*d*_, *k*_*1*_, and *k*_*2*_ for period-maintained case (red) were 637, 328, 327, 663, 311, and 601, respectively. Corresponding values for period-shortening case (black) were 570, 594, 597, 615, 354, and 303, respectively, while the other parameters were fixed for the simplicity (see text). Equations were solved numerically using the Runge–Kutta method with Δ*t* = 0.01.

The dynamics of the negative feedback model are shown in Fig 2A. The first term on the right hand side of the equation (Fig 2A) denotes the expression of a clock gene that is downregulated by a nuclear clock protein (*P*) with cooperativity (*n*). For certain parameter choices, limit cycle oscillations are produced by the model. In this study, we assume that all biochemical reaction speeds increase with temperature. In the present model, reaction speeds as represented by *v*_*S*_, *v*_*M*_, *v*_*D*_, *k*_*S*_, *k*_*1*_, and *k*_*2*_ are assumed to increase with temperature while parameters such as Michaelis constants (*K*_*I*_, *K*_*M*_, *K*_*D*_) and the Hill constant (*n*) are fixed for simplicity. Using this negative feedback model, we numerically analyzed the conditions for period stability with changing temperature. We analyzed the conditions with multiple parameter sets as the results can be parameter dependent. The analysis conditions are as follows: (1) we randomly prepared “basal” parameter sets (500 sets) for oscillations and then (2) we randomly increased all reaction speeds from the basal parameter sets (100 sets for each basal parameter set). The ratios of increased speeds to basal speeds were set to range uniformly from 1.5 to 2.5. Thus, the ratios of increased speed to basal speed for the degradation of mRNA (*v*_*M*_) and that of proteins (*v*_*D*_) can differ (for example, 2.1 and 1.9, respectively).

With the increased reaction speeds, the periods tend to be shorter than the original periods with basal parameter sets (Fig 2B and 2C). Calculated oscillations are often out of the range of temperature compensation (0.85<*Q*_10_<1.15). When oscillations are nearly temperature compensated and the periods are relatively unchanged from the original periods, the amplitudes of mRNA (*M*) oscillations always exceed the original amplitudes yielded by the basal sets (Fig 2C and 2D). Moreover, when the periods are relatively unchanged, not only the mRNA amplitudes (*M*) but also the amplitudes of other variables (*R*, *P*) tend to increase simultaneously. To measure amplitude changes of all variables, we used the geometrical mean amplitude ratio while it is possible that other metrics could be better. The numerical results of the negative feedback model suggest that temperature–amplitude coupling of some clock genes (Fig 1) can cause period stability with temperature.

### How does temperature–amplitude coupling stabilize circadian period under changing temperature?

In contrast to the negative feedback model described above, circadian rhythms in mammals and *Drosophila* involve both positive and negative feedback loops [24,31]. To understand the mechanisms for period stability in networks with under both types of feedback, we developed a full model with both positive and negative feedback loops and then simplify it to a two-variable model (S1 Text). The simplified dynamics are:

$$\varepsilon dX/dt=\left(k+vX^{\gamma }/\left(K_{V}^{\gamma }+X^{\gamma }\right)\right)/\left(h+Y\right)-aX$$ (1)

$$dY/dt=sX^{\alpha }/\left(K_{S}^{\alpha }+X^{\alpha }\right)-dY$$ (2)

where *X* is mRNA and *Y* is inhibitor protein. The first term on the right-hand side of Eq (1) indicates negative feedback regulation by an inhibitor protein (*Y*) and indirect positive feedback regulation. We assume cooperativity (*γ*) for positive feedback regulation. Production of inhibitor is also a nonlinear function of mRNA concentration (*X*) and accumulation of inhibitor (*Y*) is assumed not to occur immediately after the transcription. Degradation rates of mRNA and protein are expressed by linear terms for simplicity. Again, we assume that all the reactions represented by rates in linear kinetics (*a*,*d*) and maximum rates in Michaelis–Menten kinetics (*v*, *k*, *s*) become faster with increasing temperature, while all other parameters are fixed. We then examined the role of oscillation amplitude in period stability under these conditions.

**Fig 3:**
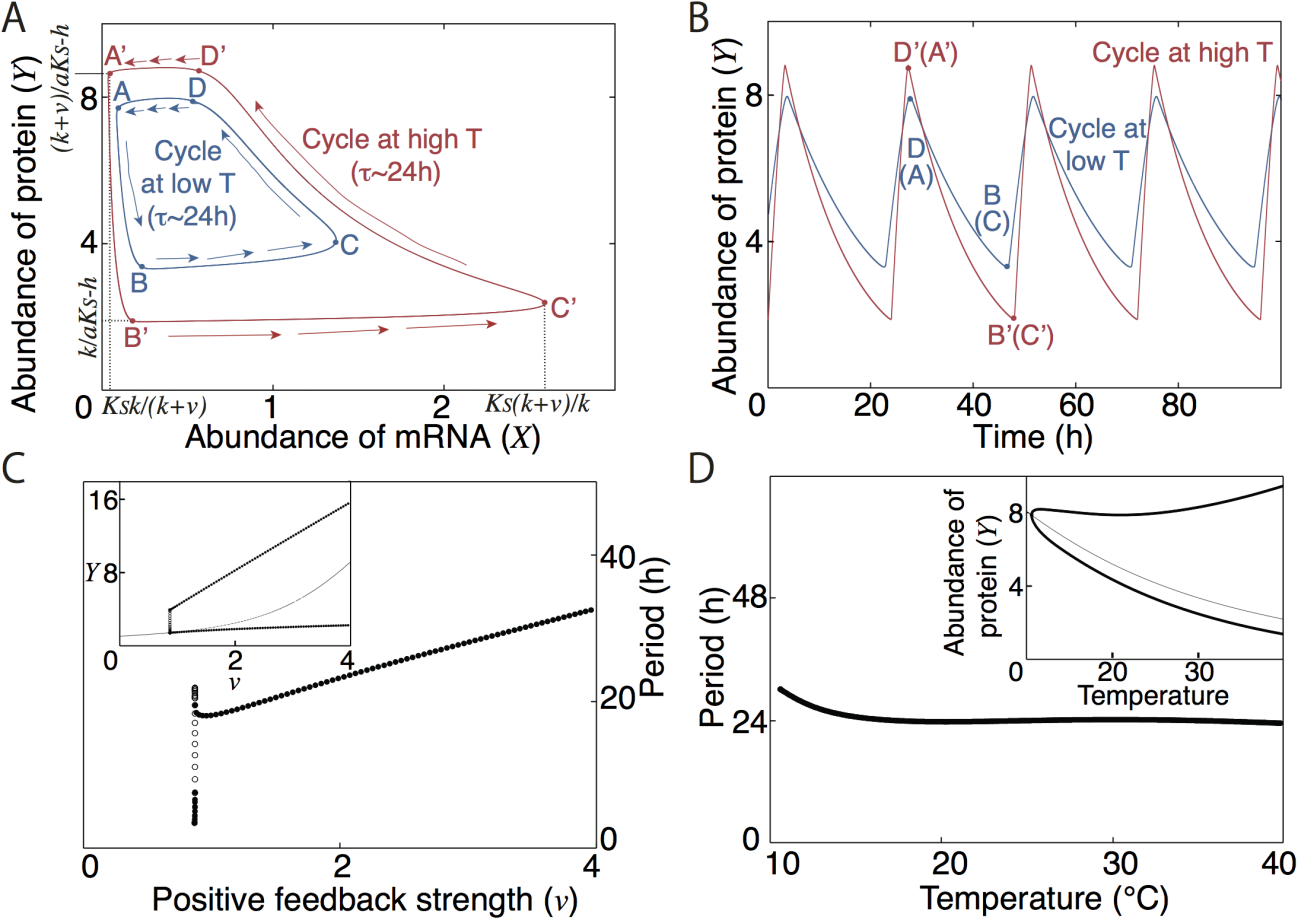
Amplitude adjustment for temperature compensation in a two-variable model. (A,B) Oscillatory orbits (A) and time series of protein (B) at higher temperature (with higher reaction speeds) and at lower temperature (with lower reaction speeds). Parameters are *k* = 0.0625, *a* = 0.0923, *v* = 0.529, *s* = 1.48, and *d* = 0.0507 for lower temperature and *k* = 0.0976, *a* = 0.302, *v* = 2.15, *s* = 2.80, and *d* = 0.0778 for higher temperature in Eqs (1) and (2), while the other parameters *h* = 0.5, *K*_*v*_ = 0.5, *K*_*S*_ = 0.5, *α* = 4, *γ* = 4, and *ε* = 0.02 are fixed. (C) Period and amplitude (*Y*) sensitivities as a function of the positive feedback regulation rate *v*. Other parameters were fixed to values for higher temperature as in (A). Small-amplitude oscillations occur through a supercritical Hopf bifurcation at *v* = 0.866. As *v* increases, amplitude of oscillation sharply increases. (D) Period and amplitude sensitivities with increasing temperature, for which we increased all the reaction rates *k*, *b*, *a*, *s*, and *d* by different ratios. Activation energies (*E*_*i*_) of *k*, *v*, *a*, *s*, and *d* are 335, 1057, 893, 480, and 322, respectively. With this setting, two examples at lower temperature (T = 298K) and higher temperature (T = 308K) are depicted in (A). Eqs (1) and (2) were solved numerically by the Runge–Kutta method with Δ*t* = 0.01 using XPPAUT [34].

Given that our model is simplified to a two variables, we can derive an approximate formula for the period by assuming that the dynamics of the activator (*X*, mRNA) is sufficiently faster than that of the inhibitor (*Y*, protein). The period formula and the technical details of the mathematical derivations are presented in S1 Text [32]. Using the equation for period, period can be coupled with amplitude. If all the rate parameters are multiplied by a common constant, the period shortens by a factor equal to the inverse of that constant [9,33], meaning that in general the period tends to shorten along with increasing temperature and reaction speed. In contrast, the positive feedback strength (*v*) always lengthens the period in this model. As positive feedback strength (*v*) increases, the peaks of *X* and *Y* rise and the nadir of *X* decreases: thus, the amplitudes of *X* and *Y* always become larger (Fig 3A–3C, S1 Text). This increase in amplitude results in period-elongation. These results suggest that a period-increasing reaction can also be an amplitude-increasing reaction. If the temperature sensitivity of an amplitude-increasing reaction is stronger, and amplitude is larger at higher temperature, the period can be stable with temperature due to cancelation of the period-shortening effect (Fig 3D). Further, we can consider the necessity of temperature-amplitude coupling for this simple model (S1 Text). Using the period formula for a certain parameter condition (i.e. *ds/dT>dd/dT* in which *T* is temperature), we can mathematically prove that it is impossible to maintain the period along with increasing temperature and reaction speeds if maximums of variables at higher temperature are smaller than those at lower temperature, and minimums of variables at higher temperature are larger than those at lower temperature. Therefore, larger maximum or smaller minimum at higher temperature is necessary for temperature compensation, qualitatively consistent with the numerical results of Goodwin model and experimental results.

### Temperature–amplitude coupling can stabilize period in a detailed model of the mammalian circadian clock

**Fig 4:**
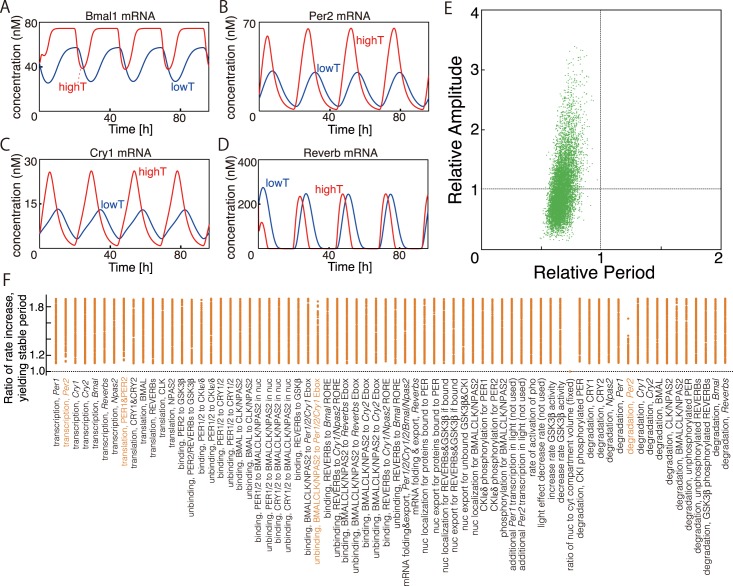
Sensitivities of period and amplitude to temperature in a detailed mammalian circadian model [30]. (A-D) Temperature-dependent time series of oscillatory variables for the model proposed by Kim and Forger (2012). We used the original parameter set described [30] and randomly increased the reaction rate parameters. Here we show examples of calculated time series for *Bmal1*, *Per2*, *Cry1*, and *Reverb* mRNAs at high (red) and low temperature (blue). Parameter values are listed in S1 Text. (E) Distribution of geometric mean of all relative amplitudes (180 variables) as a function of relative period for 2,495 parameter sets as reaction rates are increased. We randomly increased parameter values from 1.1- to 1.9-fold and generated a total of 10,000 parameter sets. The average parameter increase was ~1.5-fold. Of these sets, oscillations are sustained for 2,495. For each set, we plot the distribution of period change. (F) Variation in the parameter sets yielding relatively stable period (ratio of the new period to the original period >0.8). We assume that the parameter for “volume ratio between cytosol and nucleus (*Nf*)” is not sensitive to temperature and so was fixed. Ordinary differential equations were solved numerically using the Euler method with Δ*t* = 0.001.

The regulatory network for the mammalian circadian clock is more complex than the models described thus far, so we examined if the amplitude of oscillation is also important for period maintenance in the more detailed mammalian circadian clock model proposed by Kim and Forger (2012). Using the original parameter set [30], we assume that all the reactions in the system (with 70 parameters) become faster as the temperature increases except for the ratio of nuclear to cytosolic compartment volume (Fig 4A–4F).

In the calculations, the rates were randomly increased 1.1–1.9 fold, generating 10,000 parameter sets, and the sensitivities of period and amplitude to temperature were assessed (Fig 4E and 4F). Of the 10,000 parameter sets, oscillations are maintained for 2,495 sets. For other sets, the time series of the system converge to equilibria or do not converge to any periodic solutions after a certain calculation time. When oscillations are maintained with faster reactions, the period tends to shorten (Fig 4E). When periods are relatively maintained, the geometric means of the amplitudes of 180 variables tend to increase simultaneously, in agreement with the theoretical results from the simpler models described in this article. It appears that relative periods (new periods with faster reactions, divided by original periods) are always smaller than unity. This result does not quantitatively correspond to our experimental results showing that *Q*_10_ values of circadian mRNA oscillation periods are close to or larger than unity (Fig 1). When the rates were randomly increased within a wider range (i.e. 1.1- to 5-fold), the temperature-compensated oscillations could be reproduced in which amplitude of *Cry*1 and *Per2* are larger at higher temperature. Example of time series is depicted in Fig 4A–4D. The calculated period at high and low temperatures are 24.4 and 23.9h, respectively. This result suggests the possibility that some reactions of mammalian circadian rhythms are highly temperature-sensitive.

Among the parameter sets that can achieve relatively stable periods, some successfully yield time series of *Per2*, *Bmal1*, *Rev-erbα*, and *Cry1* mRNA expression levels in good qualitative agreement with actual profiles from mammalian cells in which amplitude of *Cry*1 and *Per2* are larger at higher temperature (Figs 1 and 4A–4D). Thus, temperature–amplitude coupling of *Cry1* oscillations as observed in our experiments (Fig 1) is indeed one possible solution for period stability in the detailed model (yielding a temperature-compensated period). From this calculation, we can also identify the essential reactions for temperature compensation in the mammalian circadian clock system. Among the 2,495 parameter sets that yielded sustained oscillations, the period is relatively stable, with relative period > 0.8, for 176 parameter sets (Fig 4F). Although many different parameter sets yield a relatively stable period, some general necessary conditions can be identified. In Fig 4F, we plot the parameter sets that yield relatively stable periods (176 sets). Notably, a stable period does not occur if degradation of *Per2* mRNA or unbinding rate of BMAL–CLK complexes or NPAS2 proteins to *Per1/2/Cry1* Ebox is greatly increased (i.e., due to high temperature sensitivity). If degradation of *Per2* mRNA is weakly temperature sensitive and its synthesis is strongly temperature sensitive, the amplitude of *Per2* mRNA oscillation should increase with temperature. Similarly, if the unbinding rate of BMAL–CLK complexes or NPAS2 proteins from *Per1/2/Cry1* Ebox is weakly temperature sensitive but binding rate is strongly temperature sensitive, *Per1*, *Per2*, and *Cry1* mRNA expression levels should increase with temperature, in good agreement with the data (Fig 1). From this analysis of the detailed model, we conclude that temperature–amplitude coupling is a plausible mechanism for period stability with temperature that holds not only for simple models but also for a more realistic detailed model.

### Period stability and amplitude of yeast respiration rhythms

In yeast, glycolytic cycles with periods on the order of minutes are sustained under a constant environment, but both period and amplitude decrease with temperature [35]. Tu et al. (2005) reported that an autonomous YMC with period of approximately 4 h occurs after deprivation of nutrients, and this period is temperature compensated [5,36]. To validate the contribution of temperature–amplitude coupling in period stability with temperature, we revisited the time series of YMC, which is Fig 1D (http://www.tandfonline.com/doi/abs/10.4161/cc.6.23.5041) of Chen and McKnight (2007) [5]. It was shown that the oscillator period measured from a time series of dO_2_ is relatively unchanged over a temperature range from 25°C to 35°C. Notably, as temperature increases from 25°C to 35°C, the nadir of the oscillations decreases and the amplitude increases. Conversely, the amplitude decreases with temperature from 35°C to 25°C, suggesting that the high amplitude sensitivity to temperature is reproducible. Although the molecular mechanisms underlying YMC are unknown, the present experimental and theoretical results suggest that temperature sensitivity of gene expression amplitude accounts for the maintenance of period under temperature changes.

## Discussion

How can the period of circadian oscillations be maintained under temperature variation when the underlying reactions accelerate with temperature [6–21]? By analyzing the sensitivities of circadian clock period and amplitude to temperature change in mammalian cultured cells, we found temperature–amplitude coupling of *Cry1* and *Dbp* mRNA oscillations. We then addressed the question of whether this observed temperature–amplitude coupling can cause temperature compensation by testing various plausible circadian models with different network topologies. For all the models we studied, when the periods with faster reaction speeds are relatively unchanged from the original periods, the geometrical mean amplitudes always exceed the original amplitudes yielded by the basal sets. These numerical results indicate that larger amplitudes with faster reaction speeds are necessary for stable period for the parameters we tested. Moreover, using our two-variable model, we mathematically showed that larger maximum or smaller minimum at higher temperature is needed for stable period for a certain parameter condition. These theoretical results suggest that temperature–amplitude coupling, observed experimentally (Fig 1) should be necessary for temperature compensation. Our results are consistent with the result of Lakin-Thomas et al. (1991), who showed that temperature–amplitude coupling can lead to a stable period in one-variable model with delay [7].

Previously, temperature compensation of circadian rhythms has been studied by quantifying emission generated by a bioluminescence reporter [37], melatonin secreted in a culture medium [38], and neural firing [39] as well as gene expression [2, 37, 40,41]. While the range of temperature compensation has been defined as 0.85<*Q*_10_<1.15, longer period at higher temperature was reported by many literatures, so called "temperature overcompensation" [2, 37, 38,40,41]. Temperature overcompensation is within the range of temperature compensation. In consistent with those previous reports, quantification of gene expression in the present study showed that the circadian period of C6 glioma cells is longer at higher temperature (Fig 1). We also confirmed this temperature overcompensation by using a cell line expressing *Bmal1*::*luc* (*Q*_10_ = 0.86. S1 Fig). In contrast, numerical simulations in the present study showed that it is difficult to achieve perfect temperature compensation (*Q*_10_ = 1) or temperature overcompensation (*Q*_10_<1) when reactions speeds are assumed to increase 2–3 fold or 1.1–1.9 fold with 10°C temperature rise (Figs 2C and 4E). When reactions speeds are assumed to increase within a wider range (i.e. 1.1–5 fold), temperature compensation is more likely to occur (Fig 4A–4D). This numerical result suggests the presence of strongly temperature sensitive reactions in circadian rhythms for temperature compensation and overcompensation.

In some previous experimental studies, amplitude of circadian oscillations was reported to decrease with temperature [6,40,42], which is inconsistent with the conclusion of the present study. We think that this discrepancy might be due to temperature sensitivity of bioluminescence although we cannot exclude the possibility that temperature-amplitude coupling does not hold for those rhythms. The intensity of bioluminescence increases at lower temperatures, peaks around 25°C, and drops sharply at higher temperatures [43]. This indicates that the bioluminescence level does not proportionate to the amount of the gene expression when temperature was being changed. In our present experiments, the amplitude of the bioluminescence level of *Bmal1*::*luc* decreased while the level of the gene expression of *Bmal1* mRNA increased along with the rise of the temperature (Fig 1, S1 Fig).

Notably, the expression amplitudes of some genes (e.g., *Rev-erbα*) in mammalian cultured cells did not change with temperature while those of others (e.g., *Cry1* and *Dbp*) increased markedly with temperature. From the theoretical results of models with different network topologies (S1 Text, S2 Fig), we can interpret temperature-dependent amplitudes of some transcripts (e.g., *Cry1* and *Dbp*) and temperature-independent amplitudes of the other transcripts (e.g., *Rev-erbα*) as a consequence of a network structure of circadian rhythm with positive feedback loop(s).

Ferrell and coworkers found that models with both negative and positive feedbacks can yield a various period with fixed time series amplitudes depending on the specific parameter values [44,45]. These studies suggest that period stability to temperature is independent of amplitude when the network includes positive feedback. Unexpectedly, we found that the period tends to be maintained when the geometric mean amplitude increases with reaction speed for both models with and without positive feedback (S1 Text; S2 Fig). We think that the discrepancy between our results and a previous theoretical study [45] is due to differences in model structure or our parameter settings. In our models, the effects of temperature are incorporated, all the reactions are assumed to increase in speed with temperature, and the parameters are often far from bifurcation points.

In this study, we analyzed transcriptional–translational feedback loop models. In contrast, the temperature compensation of *cyanobacteria* circadian rhythms was suggested to be generated by a post-translational network or by a single key protein (KaiC) [11,46,47]. It is possible that these mechanisms also hold for temperature compensation of mammalian circadian rhythms [13,14]. Indeed, circadian oscillations are maintained even when transcription or translation is greatly suppressed in mammalian peripheral cells [13,48], and previous works have shown the role of phosphorylation for temperature compensation [14,19,49]. Although temperature compensation can still occur in our regulatory network model described by Eqs (1) and (2) with partial inhibition of transcription or translation (S3 Fig), we cannot exclude the possibility that temperature compensation in mammalian peripheral cells is generated by post-translational network or by a single key reaction. Notably, in the report of Nakajima et al. (2005), the rhythm amplitude of cyanobacterial KaiC protein phosphorylation increased with temperature. We suggest that it is important to study the theoretical possibility that temperature–amplitude coupling can lead to period stability under temperature variation even in post-translational network models [21].

Is temperature–amplitude coupling a general mechanism for temperature compensation? Although we do not have an answer to the question, we found that temperature–amplitude coupling stabilizes the period for all models studied despite differences in network topology. Furthermore, we confirmed that respiration rhythm amplitudes also increase with temperature in the (temperature-compensated) YMC [5]. Previously, Ruoff demonstrated that balance between period-increasing and -decreasing reactions can generate temperature compensation in any biochemical oscillators [8,50] which was supported by recent experiments of circadian rhythms in *Neurospora* [9] and mammals [19]. This balance theory and temperature–amplitude coupling hypothesis are not mutually exclusive, and we think that these are connected. In fact, mathematical analysis of our simpler model showed period-increasing reaction can be simultaneously amplitude-increasing reaction. This result suggests that balance between period-increasing and -decreasing reactions works through modulating oscillator amplitude for achieving temperature compensation. Our prediction of temperature–amplitude coupling can also be tested in a different way using phase response curve experiments [7]. Ute et al. (2010) reported that the range of entrainment increases as the ratio between zeitgeber strength and oscillator amplitude increases, suggesting that the magnitude of the phase response curve (PRC) to certain stimuli increases as oscillator amplitude decreases [51]. If oscillator amplitude is larger at higher temperature, then the magnitude of PRC in response to stimuli at higher temperature should be smaller than that at lower temperature. Indeed, the magnitude of the phase shift by light was smaller at higher temperature in *Neurospora* [52]. Recently, the magnitude of the phase shift by light was shown to be smaller (type 1) at higher temperature than at lower temperature (type 0) in *Drosophila* [53]. Measurements of PRC at different temperatures can also be conducted in a circadian mammalian culture system or yeast respiration rhythms system modulated by chemical stimuli (such as forskolin or H_2_O_2_, respectively) [54]. Such experiments could reveal similarities or differences in the mechanisms underlying period stability to temperature between circadian rhythms and yeast respiration rhythms.

## Materials and methods

### Cell culture and real-time monitoring of circadian bioluminescence

C6 glioma cells and C6-*Bmal1*::*dluc* cells (kindly gifted by Dr. Kazuhiro Yagita) were plated on 35 mm dishes and cultured in DMEM containing 10% FBS for several days at 37°C. After the cells had reached confluence, they were incubated in serum-free DMEM for a further 24 h and then separately stimulated by supplementation with 100 nM dexamethasone (Dex, ICN). After 2 h of treatment with Dex, the culture medium was replaced with serum-free DMEM and the dishes were moved to the incubator or of 38°C or 35°C in a 5% CO_2_ atmosphere. For bioluminescence measurements (S1 Fig), the dishes were moved to photomultiplier tube (PMT), detector assemblies (Kronos Dio, ATTO, Tokyo, Japan) with same temperature and CO_2_ condition described above. To analyze period of bioluminescence, detrended traces computed by a software supplied with PMT, were fitted to an exponentially damped sine curve y = y0 + Ae^(−x/t0)^sin[π(x − x_c_)/w] using software (Origin8.1J; OriginLab, MA, USA) [55]. To determine the significance of the differences, Student’s *t*-test was used (^***^ *p* < 0.001).

### Quantitative PCR

From 32 h to 96 h after the treatment with Dex, cultured cells (*n* = 3) were harvesting 800 μl nucleic acid purification lysis solution (ABI) every 4h and then total RNA was extracted with an ABI Prism 6100 Nucleic Acid PreStation (ABI). An 0.5 μg aliquot of each total RNA preparation was then reverse transcribed using ReverTra Ace (Toyobo) and 2.5 μM oligo (dT). TaqMan real-time PCR (qPCR) was next performed with an ABI PRISM 7700 Sequence Detector, in a total volume of 15 μl using Premix Ex Taq (Perfect Real Time) (Takara), according to the supplier’s instructions. mRNA quantification was performed with two primers and a fluorescent probe as follows: r*Bmal1*: forward primer, CTGAG CTGCCTCGTTGCA; reverse primer, CCCGTATTTCCCCGTTCACT; probe, TCGGGCGACTGCACTCACACATG; *rCry1*: forward primer, TTCGTCAGGAGGGCTGGAT; reverse primer, GCCGCGGGTCAGGAA; probe, CACCATCTAGCCCGACATGCAGTTGC; r*Dbp*: forward primer, TGCCCTGTCAAGCATTCCA; reverse primer, AGGCTTCAATTCCTCCTCTGAGA; probe, TCGACATAAAGTCCGAACGAGCCCG; r*E4bp4*: forward primer, CAGGTGACGAACATTCAAGATTG; reverse primer, TTGCCGCCCAGTTCTTTG; probe, TCCCTCAGATCGGAACACTGGCATC; r*Per2*: forward primer, GCTCTCAGAGTTTGTGCGATGA; reverse primer, AAAAGACACAAGCAGTCACACAAATA; probe, TTGTTCATGCG CAAACCAAACGTACC; r*Per3*: forward primer, CCGGAAGGTCTCCTTCATCAT; reverse primer, TGGTGGCAAAAACATCTTCATT; probe, TCGACATAAAGTCCGAACGAGCCCG; and r*Rev-erbα* (Nr1d1): forward primer, TGAAAAACGAGAACTGCTCCATT; reverse primer, CCAACGGAGAGACACTTCTTGAA; probe, TATCAATCGCAACCGCTGCCAGC; and. For the normalization of template concentrations, primers and a probe for glycaldehyde-3-phosphate dehydrogenase (GAPDH (ABI)) were used. The resulting threshold cycle (Ct) values from the cDNA amplifications were thus normalized to the Ct values for GAPDH. With these results, the period and amplitude of each gene expression were analyzed with ARSER [25], which supplied the period and amplitude of the circadian rhythm. The experiment was repeated three times independently and, by applying ARSER, amplitude and period for each experiment were obtained and statistically analyzed for the effect of temperature change. To determine the significance of the differences, Student’s *t*-test was used (^*^ *p* < 0.05 and ^**^ *p* < 0.01).

### Computations

Ordinary differential equations are solved numerically by Runge-Kutta method with Δ*t* = 0.01 except for a detailed mammalian circadian model. For the detailed model, we used the Euler method with Δ*t* = 0.001. To express temperature sensitivity in rate constants for Fig 2D, Fig 3D and S3 Fig, we used Arrhenius relation for each reaction rate. We obtained period and amplitude sensitivity to temperature or reaction rates (Fig 3, S3 Fig) by the Runge-Kutta method with Δ*t* = 0.01 using XPPAUT [34]. Relative amplitude in Figs 2 and 4, and S2 and S4 Figs was defined as the new amplitude with increased reaction speeds divided by the basic period with basic parameter sets.

## Supporting information

S1 Text Computation and mathematical analyses of clock models. (PDF)

S1 Fig Temperature-dependent luminescence rhythm of C6-*Bmal1*::*dluc* cells. (TIF)

S2 Fig Amplitude adjustment for temperature compensation in multiple models with different network topologies. (TIFF)

S3 Fig Period as a function of temperature when transcription or translation is inhibited 20%. (EPS)

S4 Fig Amplitude adjustment for temperature compensation in sequestration model. (EPS)
