# Supplementary material for: Temperature–amplitude coupling for stable biological rhythms at different temperatures
Source: PLoS Comput Biol. 2017 Jun 8;13(6):e1005501. doi: 10.1371/journal.pcbi.1005501 (PMC5464531; doi:10.1371/journal.pcbi.1005501)
Supplement: S1 Text — (PDF) [file pcbi.1005501.s001.pdf]

S1 Text. Computation and mathematical analyses of clock models

Temperature-amplitude coupling can stabilize period at multiple models with different network topologies

In reality, both positive and negative feedback loops underlie circadian rhythms [24,31]. Our results for the Goodwin model with only negative feedback are consistent with Ferrell and co-workers [45], which showed that when the period is changed by parameter variation, the amplitude is also changed in a model with only negative feedback. They also found that the period can be tuned without amplitude changes in a model with both negative and positive feedback [45]. Their findings raise the possibility that the condition for period stability to temperature change depends on network topology. To investigate this possibility, we considered the following two hypothetical models with different network structures: (i) a positive plus negative feedback model and (ii) a negative plus negative feedback model. The structures of the networks and equations for the two models are presented in S2A and S2B Fig. A positive regulation term in the form $vM^{\gamma}/\left(K_v^{\gamma}+M^{\gamma}\right)$ is added to the dynamics of mRNA (M) of the Goodwin model in (i) to yield the positive plus negative feedback loop model. Negative regulation in the form of $v/\left(K_v^{\gamma}+M^{\gamma}\right)$ is added to the dynamics of mRNA (M) in (ii) to yield the negative plus negative feedback model. The dynamics of the added regulations are assumed to be sufficiently fast that intermediate steps needed to generate an activator (S2A Fig) or inhibitor (S2B Fig) are negligible.

Does the period sensitivity to rate increase differ between the positive plus negative and the negative plus negative feedback models? To investigate this question, all reaction speeds, including rates in linear kinetics (k_S, k_1, k_2) and maximum Michaelis–Menten rates (v_S, v_M, v_D, and v) in the two models were randomly increased. Again, we set the ratios of increased speeds to basal speeds uniformly from 1.5 to 2.5. Period is more likely to change with faster rates in the positive plus negative feedback model than in the negative plus negative feedback model (S2C and S2D Fig). In both models, the period tends to be maintained when the amplitude of mRNA (M) at faster rates exceeds the original amplitude yielded by basal parameter sets (pink dot, S2E and S2F Fig). Notably, the period can also be maintained without change in mRNA amplitude (M) for some parameter sets in the positive plus negative feedback model, but

not in the negative plus negative feedback model. These dependencies of period and amplitude on network topology (pink, dot S2E and S2F Fig) are consistent with the abovementioned results of Tsai et al. (2008) showing tunability of period without change in amplitude in models with positive feedback loop(s). Unexpectedly, in both of our models (S2A and S2B Fig), the period was maintained with increasing reaction speeds when the geometric mean of three amplitudes at faster rates exceeded the original geometric mean at basal speeds (green dot, S2E and S2F Fig). We also found that the positive plus negative feedback model is more likely to stabilize the period along with increasing reaction speeds. When the period was measured numerically under the increase of kinetic parameter one by one by 1.1 fold, period often shortened (S2G). In contrast, faster positive feedback regulation, transport to cytosol, and transcription caused longer period, suggesting that effects of these reactions cancel out the period-shortening effect to stabilize period.

Taken together, these results show that larger geometric mean amplitudes (due to faster reactions) can lead to period stability in models with different network topologies. Using these theoretical results, we can interpret the temperature-dependent amplitudes of some transcripts (e.g., *Cry1* and *Dbp*) and temperature-independent amplitudes of other transcripts (e.g., *Rev-erbα*) as a consequence of the network structure with positive feedback loop(s). Moreover, our results (S2 Fig) suggest that temperature–amplitude coupling of many genes (Fig 1) results in larger geometric mean amplitudes that stabilize the circadian period under changing temperature.

Temperature-amplitude coupling can stabilize period at sequestration model

Recently, protein-sequestration type function rather than Hill-type function has been also used to express transcription repression in circadian clock models [30,56]. To test the function dependency of the condition for temperature compensation, we study the following model [56]:

$$\begin{aligned} dM/dt &= \alpha_M f(A,R) - \beta_M M, \\ dC/dt &= \alpha_C M - \beta_C C, \\ dR/dt &= \alpha_R C - \beta_R R, \end{aligned} \qquad \text{(A1)}$$

in which $f(A,R)=\left(A-R-k_D+\sqrt{\left(A-R-k_D\right)^2+4Ak_D}\right)\Big/2A$. Using this model and the original parameter set [56], sensitivity of period to increasing reactions speeds was numerically analyzed as we did in the previous sections. In the sequestration model, the period can be close to the range of temperature compensation without the change in mRNA amplitude (*M*) for some parameter sets (S4 Fig). However, it appeared that the period was relatively maintained with increasing reaction speeds when the geometric mean of three amplitudes at faster rates exceeded the original geometric mean at basal speeds also in this model, indicating that temperature-amplitude coupling can stabilize the period in the sequestration model.

Derivation of a simpler model

In the following, a two-variable model for circadian rhythm (Equation (1) and (2) in the main text) is derived. Based on mammalian regulatory gene network [24], we first model the full dynamics of mRNAs and proteins for the clock genes of *Per*, *Cry, Bmal*, and *Rorc*. Expression levels of the core clock genes *Per* and *Cry* are activated by BMAL–CLK complex and inactivated by PER–CRY complex through E-box. In addition, we incorporate dynamics of RORC, which activates the expression of *Bmal* through the RORE element (although the *Ror* gene is not necessary for the generation of cellular rhythms because positive feedback through RORC is expected to enhance the occurrence of oscillations) [24]. We neglect the dynamics of REVERB and CLK for simplicity. The dynamics are expressed as follows:

$$dP_M/dt=\left(k_P+vB_P^\gamma\Big/\left(K_v^\gamma+B_P^\gamma\right)\right)\Big/\left(h+C_PP_P\right)-a_PP_M, \quad \text{(A2a)}$$

$$dC_M/dt=\left(k_C+vB_P^\gamma\Big/\left(K_v^\gamma+B_P^\gamma\right)\right)\Big/\left(h+C_PP_P\right)-a_CC_M, \quad \text{(A2b)}$$

$$dR_M/dt=\left(k_R+vB_P^\gamma\Big/\left(K_v^\gamma+B_P^\gamma\right)\right)\Big/\left(h+C_PP_P\right)-a_RR_M, \quad \text{(A2c)}$$

$$dB_M/dt=k_BR_P-a_BB_M, \quad \text{(A2d)}$$

$$dP_P/dt=s_PP_M^{\alpha_P}\Big/\left(K_{SP}^{\alpha_P}+P_M^{\alpha_P}\right)-d_PP_P, \quad \text{(A2e)}$$

$$dC_P/dt=s_CC_M^{\alpha_C}\Big/\left(K_{SC}^{\alpha_C}+C_M^{\alpha_C}\right)-d_CC_P, \quad \text{(A2f)}$$

$$dR_P/dt=s_RR_M-d_RR_P, \quad \text{(A2g)}$$

$$dB_P/dt = s_B B_M - d_B B_P. \quad \text{(A2h)}$$

In the model, *Per*-mRNA, *Cry*-mRNA, *Rorc*-mRNA, *Bmal*-mRNA, PER protein, CRY protein, RORC protein, and BMAL protein correspond to P_M, C_M, R_M, B_M, P_P, C_P, R_P, and B_P, respectively. Degradations rates of mRNAs and proteins are expressed by linear functions. Activation of *Bmal* gene expression by RORC is also expressed by a linear function for simplicity. To derive a simpler model of Equation (1) and (2), we use three assumptions: (i) given that transcription levels of *Per*, *Cry*, and *Rorc* are regulated through Ebox, the dynamics of *Per*, *Cry*, and *Rorc* mRNAs can be expressed by a single variable for simplicity (named X; $X=P_M=C_M=R_M$); (ii) dynamics of PER and CRY proteins can be expressed by a single variable (named Y; $Y= P_P=C_P$) for simplicity, in which the negative feedback regulation by a complex of PER and CRY is expressed as a nonlinear function of Y with cooperativity (n); (iii) the dynamics of transcription as well as post-transcriptional regulation of *Bmal* and *Rorc* are faster than the dynamics of transcription and post-transcriptional regulation of *Per* and *Cry*. Using assumptions (i) and (ii), Equations (A2a–h) become:

$$dX/dt = \left(k + v' B_P^{\gamma} \big/ \left(K_v^{\gamma} + B_P^{\gamma}\right)\right) \big/ \left(h + Y^n\right) - aX, \quad \text{(A3a)}$$

$$dB_M/dt = k_B R_P - a_B B_M, \quad \text{(A3b)}$$

$$dY/dt = sX^{\alpha} \big/ \left(K_S^{\alpha} + X^{\alpha}\right) - dY, \quad \text{(A3c)}$$

$$dR_P/dt = s_R X - d_R R_P, \quad \text{(A3d)}$$

$$dB_P/dt = s_B B_M - d_B B_P. \quad \text{(A3e)}$$

For assumption (iii), using the steady-state approximations of $dR_P/dt \approx 0$, $dB_P/dt \approx 0$, and $dB_M/dt \approx 0$, we obtain $B_P = s_B B_M / d_B = s_B k_B R_P / \left(d_B a_B\right) = s_B s_R k_B X / \left(d_B d_R a_B\right) \propto X$ for which the abundance of Bmal1 protein (B_P) in Equation A3a is replaced by the abundance of *Per*/*Cry* mRNA (X). Equations (A3a-e) are simplified into a two-variable model:

$$\varepsilon\, dX/dt = \left(k + vX^{\gamma} \big/ \left(K_v^{\gamma} + X^{\gamma}\right)\right) \big/ \left(h + Y^n\right) - aX, \quad \text{(A4a)}$$

$$dY/dt = sX^{\alpha} \big/ \left(K_S^{\alpha} + X^{\alpha}\right) - dY. \quad \text{(A4b)}$$

In Equations (A4a and A4b), a time scale constant (ε) is added to indicate the time scale of mRNAs and proteins. If the positive feedback term, $vX^{\gamma} / \left(K_v^{\gamma} + X^{\gamma}\right)$ is removed from

Equations (A4), which does not contain an explicit delay term, oscillations do not occur for any choice of parameters [57,58]. Since oscillations can occur for no nonlinearity in transcription inhibition (n=1), we fixed n=1 in our calculations.

Approximate formula for period

Using equation (A4), we derive approximate formula for period and amplitude [32]. When the dynamics of mRNA are much faster than the dynamics of protein in the model (i.e., $1/\varepsilon >> 0$, in which the dynamics of mRNA should be faster than that of protein), we can trace the limit cycle solution of $(X(t), Y(t))$ on the null-clines of the XY plane. In the following, we estimate the O(1) period of the cycle in Fig 3. We further assume (iv) that the dynamics of activator (X, mRNA) are sufficiently faster than that inhibitor (Y, protein) (i.e., $1/\varepsilon >> 0$), (v) a high Hill coefficient for negative and positive feedback regulation (i.e., $\alpha, \gamma >> 0$), and (vi) that the threshold for the production of activator (K_V) is similar to that for the production of inhibitor (K_S). The last two assumptions may be unrealistic but are needed for the derivation of the period. Under these assumptions, the null-cline $dX/dt = 0$ can be expressed as follows:

$$\begin{aligned} Y &= \left(k + vX^{\gamma}/\left(K_v^{\gamma} + X^{\gamma}\right)\right)/aX - h \\ &\approx \begin{cases} k/aX - h & \text{for } X < K_v \\ (k+v)/aX - h & \text{for } K_v \le X \end{cases} \end{aligned} \quad \text{(A5)}$$

Similarly the null-cline $dY/dt = 0$ can be expressed as follows:

$$\begin{aligned} Y &= sX^{\alpha}/\left(d\left(K_s^{\alpha} + X^{\alpha}\right)\right) \\ &\approx \begin{cases} 0 & \text{for } X < K_s \\ s/d & \text{for } K_s \le X \end{cases} \end{aligned} \quad \text{(A6)}$$

From the expressions of the null-clines, we can obtain the coordinates (points A, B, C, and D in Fig 3A and B). Given that the value of variable X is assumed to be elevated at point B, the value of X at point B (X_B) is K_S. The value of variable Y (Y_B) is then $k/(aK_S) - h$ from Equation (A5). At point D, the value of X is assumed to be the same as the value of X at point B. The value of X (X_D) at point D is then K_S. From Equation (A4), the value of variable Y at point D (i.e., the maximum of Y, Y_D) is $(k+v)/aK_S - h$. We assume that the time scale of the change in variable X is much faster than that of

variable Y, so the value of variable Y at point A is approximately the same as that of Y at point D. Thus, Y_A=Y_D=$\left(k+v\right)/\left(aK_S\right)-h$. Similarly, the value of Y at point C is approximately same as that of Y at point B. Thus, Y_C=Y_B= $k/\left(aK_S\right)-h$. Taken together, (X_A,Y_A)=($kK_S/\left(k+v\right)$,$\left(k+v\right)/\left(aK_S\right)-h$), (X_B,Y_B)=(K_S, $k/\left(aK_S\right)-h$), (X_C,Y_C)=($K_S\left(k+v\right)/k$, $k/\left(aK_S\right)-h$), and (X_D,Y_D)=(K_S, $\left(k+v\right)/\left(aK_S\right)-h$). By calculating the time taken to pass between these points (i.e., A, B, C, and D), we can estimate the period. The time between the points A and B is

$$T_{AB}=\int_A^B dt=\int_{Y_A}^{Y_B}\left[dY/dt\right]^{-1}dY\ . \qquad \text{(A7)}$$

From the coordinates of Y_A and Y_B, and approximate expression of dY/dt=0,

$$\int_{Y_A}^{Y_B}\left[dY/dt\right]^{-1}dY\approx\int_{Y_A}^{Y_B}\left[-dY\right]^{-1}dY=\frac{1}{d}\log\left[\frac{\left(k+v\right)/\left(aK_S\right)-h}{k/\left(aK_S\right)-h}\right]. \qquad \text{(A8)}$$

Similarly, the time between the points C and D can be expressed as

$$T_{CD}=\int_C^D dt=\int_{Y_C}^{Y_D}\left[dY/dt\right]^{-1}dY\ . \qquad \text{(A9)}$$

Using the coordinates of Y_C and Y_D and the approximate expression of dY/dt=0,

$$T_{CD}=\frac{1}{d}\left[\log\left[\frac{s+dh-dk/\left(aK_S\right)}{s+dh-d\left(k+v\right)/\left(aK_S\right)}\right]\right]. \qquad \text{(A10)}$$

Finally, the order (1) period of the limit cycle is obtained as

$$T=T_{AB}+T_{CD}=\frac{1}{d}\left[\begin{array}{l}\log\left[\left(\left(k+v\right)/\left(aK_S\right)-h\right)/\left(k/\left(aK_S\right)-h\right)\right]\\+\log\left[\left(s+dh-dk/\left(aK_S\right)\right)/\left(s+dh-d\left(k+v\right)/\left(aK_S\right)\right)\right]\end{array}\right]. \qquad \text{(A11)}$$

Sensitivity analysis of period

Using an approximate formula for period (Equation (A11]) and amplitude, both period sensitivity and amplitude sensitivity to kinetic parameter can be examined. For instance, we consider sensitivity of amplitude and period with respect to parameter v. The amplitude of X (i.e., $vK_S\left(2k+v\right)/\left(k\left(k+v\right)\right)$) increases with v, with the X maximum increasing and X minimum decreasing. Similarly, the amplitude of Y (i.e., $v/\left(aK_S\right)$) increases with v, with the Y maximum increasing and Y minimum remaining the same. Thus, with increases in v, the amplitudes of both X and Y always increase. The

sensitivity of the period can also be examined theoretically. From (A11), the sensitivity of the period to v can be calculated as follows:

$$\partial T/\partial v = 1\big/d\left(k - ahK_s + v\right) + 1\big/\left(asK_s - d\left(k - ahK_s + v\right)\right) \quad \text{(A12)}$$

Equation (A12) is always positive because Y_D (=$\left(k+v\right)/\left(aK_S\right)-h$) is positive and because Y on dY/dt is smaller than s/d, and Y_D is smaller than s/d for generating oscillations. It implies that the positive feedback strength (v) always lengthens the period.

Condition for stable period

We can also discuss the condition of stable period to temperatures for certain parameter condition using an approximate formula for period. To discuss the condition, we first set that k',v',a',s', and d' are reaction rates at higher temperature and $k, v, a, s,$ and d are those at lower temperature, and that $k'>k$, $v'>v$, $a'>a$, $s'>s$, $d'>d$, indicating faster reaction rates at higher temperature. Then, we wish to consider whether it is possible to achieve stable period to temperature when maximums of two variables (X,Y) at higher temperature are smaller than those at lower temperature, and minimums at higher temperature are larger than those at lower temperature.

In fact, conditions for smaller X maximum, smaller Y maximum, and larger Y minimum at higher temperature correspond respectively to

$$\begin{aligned} &K_s\left(k+v\right)/k \ge K_s\left(k'+v'\right)/k' \\ &k/aK_s - h \le k'/a'K_s - h \\ &\left(k+v\right)/aK_s - h \ge \left(k'+v'\right)/a'K_s - h \\ &kK_s/\left(k+v\right) \le k'K_s/\left(k'+v'\right). \end{aligned} \quad \text{(A13)}$$

From Equation (A13), we obtain $k/a \le k'/a'$ and $v/a \ge v'/a'$. With this relation for $k,k',a,a',v,$ and v' and also with $d'>d$, we can derive

$$T_{AB} = \frac{1}{d}\log\left[\frac{\left(k+v\right)/\left(aK_S\right)-h}{k/\left(aK_S\right)-h}\right] = \frac{1}{d}\log\left[\frac{v/\left(aK_S\right)}{k/\left(aK_S\right)-h}+1\right] > \frac{1}{d'}\log\left[\frac{v'/\left(a'K_S\right)}{k'/\left(a'K_S\right)-h}+1\right] \quad \text{(A14)}$$

which indicate that T_{AB} at higher temperature is always shorter than that at lower temperature for any choice of parameter sets.

Similarly, we can analytically discuss the sensitivity of T_{CD} to temperature for a certain

parameter condition (i.e. $s'/s>d'/d$). This condition means relatively weak temperature-sensitivity for protein degradation, and corresponds to experimental fact that activation energy of protein degradation is relatively low for temperature-compensated *Neurospora* circadian clock [9]. In this condition, when maximums of variables are smaller at higher temperature and minimums of variables are larger at higher temperature, we can derive:

$$T_{CD}=\frac{1}{d}\left[\log\left[\frac{s+dh-dk/(aK_S)}{s+dh-d(k+v)/(aK_S)}\right]\right]>\frac{1}{d'}\left[\log\left[\frac{s'+d'h-d'k'/(a'K_S)}{s'+d'h-d'(k'+v')/(a'K_S)}\right]\right] \qquad \text{(A15)}$$

which indicate T_{CD} at higher temperature must be shorter than that at lower temperature. Together, we can prove that when maximums of variables are smaller at higher temperature and minimums of variables are larger at higher temperature, it is impossible to achieve stable period to temperature for the parameter condition (i.e. $s'/s>d'/d$). Therefore, larger maximum at higher temperature or lower minimum at higher temperature is necessary for temperature compensation for this setting.

Parameter values for a detailed mammalian circadian model

In Fig 4A-D, we showed time series for *Bmal1*, *Per2*, *Cry1*, and *Reverb* mRNAs at high and low temperature. We used the original parameters [30] for time series at low temperature. To reproduce longer period at higher temperature, all reaction speeds are increased 1.1- to 5-fold except for the parameter for volume ratio between cytosol and nucleus (*Nf*). The parameters used for high temperature are: *trPo*=50, *trPt*=150, *trRo*=60, *trRt*=45, *trB*=180, *trRev*=120, *trNp*=0.65, *tlp*=9.05, *tlr*=10, *tlb*=1.06, *tlrev*=17.8, *tlc*=9.2, *tlnp*=2.5, *agp*=2.7, *dg*=5.8, *ac*=0.051, *dc*=0.2, *ar*=0.047, *dr*=1.2, *cbin*=0.09, *uncbin*=14.5, *bbin*=13.8, *unbbin*=0.26, *cbbin*=13, *uncbbin*=0.6, *ag*=0.32, *bin*=14, *unbin*=0.28, *binrev*=0.024, *unbinrev*=21, *binr*=12.3, *unbinr*=5.8, *binc*=0.56, *unbinc*=0.017, *binrevb*=0.012, *unbinrevb*=10.6, *tmc*=0.32, *tmcrev*=18.5, *nl*=0.8, *ne*=0.053, *nlrev*=19.2, *nerev*=0.03, *lne*=1.18, *nlbc*=10.5, *hoo*=1.05, *hto*=4.9, *phos*=0.58, *lono*=0.4, *lont*=0.78, *lta*=1.2, *ltb*=0.026, *trgto*=1.28, *ugto*=0.069, *Nf*=3.35063, *up*=17.69, *uro*=0.34, *urt*=2.4, *umNp*=0.73, *umPo*=1.53, *umPt*=1.17, *umRo*=0.8, *umRt*=0.91, *ub*=0.037, *uc*=0.05, *ubc*=0.4, *upu*=0.14, *urev*=3.3, *uprev*=1, *umB*=2.4, *umRev*=7.5.

Supplemental References
56. Kim JK. Protein sequestration versus Hill-type repression in circadian clock models. IET Syst Biol. 2016;10(4):125-35.
57. Griffith JS. Mathematics of cellular control processes. I. Negative feedback to one gene. J Theor Biol. 1968;20:202-08.
58. Kurosawa G, Mochizuki A, Iwasa Y. Comparative study of circadian clock models, in search of processes promoting oscillation. J Theor Biol. 2002;216:193-208.

S1 Fig. Temperature-dependent luminescence rhythm of C6-*Bmal1::dluc* cells
(A) Time series of the bioluminescence intensity of C6-*Bmal1::dluc* cells at 35°C (blue) and 38°C (red). Raw (left) and detrended (right) traces are shown. (B) Periods and Q_{10} values of the circadian bioluminescence rhythm at 35°C and 38°C. Period at 38°C (23.90 ± 0.031 h, mean ± SEM, $n = 4$) was significantly longer than that at 35°C (22.84 ± 0.034 h, $n = 4$, $p < 0.001$). The period sensitivity to temperature was within the range of temperature compensation ($Q_{10} = 0.86$). Because luciferase activity is severely affected by temperature change [43], amplitudes of these circadian oscillations were not comparable.

S2 Fig. Amplitude adjustment for temperature compensation in multiple models with different network topologies. (A) Negative feedback plus positive feedback model and its equation. (B) Negative feedback with negative feedback model and its equation. (C,D) Distribution of periods when reaction rates are increased for the negative feedback model with positive feedback and the same model with negative feedback. First, starting from the standard parameter sets: n=4, v=0.2, K_V=1, γ=2, v_s=1.6, v_M=0.505, K_M=0.5, K_I=1, k_s=0.5, v_D=1.4, K_D=0.13, k_1=0.5, and k_2=0.6 in the positive plus negative feedback model (A), we prepared 500 basic parameter sets for which parameters v, v_m, k_s, v_d, k_1, k_2, and v_s are one-half to double their standard values. Second, using the 500 basic parameter sets, we constructed 100 additional parameter sets for each basic parameter set in which v, v_m, k_s, v_d, k_1, k_2, and v_s were increased randomly 1.5- to 2.5-fold above the basic parameter set values. Sustained oscillations were obtained for 31,036 of 50,000 parameter sets. We plotted the relative period), defined as the new

period with increased reaction speeds divided by the basic period with basic parameters. (E,F) The distribution of both the amplitude of oscillatory mRNA (*M*, pink) and the geometric mean (green) of the relative amplitudes of *M, R,* and *P* are plotted as a function of relative period as reaction rates are increased for the negative plus positive feedback model (E). Similarly, for the negative plus negative feedback model, we set the following standard parameter sets: n=4, v=0.1, K_V=1, γ=2, v_s=1.6, v_M=0.505, K_M=0.5, K_I=1, k_s=0.5, v_D=1.4, K_D=0.13, k_1=0.5, and k_2=0.6. We prepared 500 basic parameter sets in which v, v_m, k_s, v_d, k_1, k_2, and v_s are one-half to double their standard values, and we constructed 100 parameter sets for each basic parameter. Of 50,000 sets, 31,682 yield sustained oscillations for which we determined the frequency of the relative period (D), and plotted both the amplitude of mRNA oscillation (*M*, pink) and the geometric mean (green) of *M, R,* and *P* relative amplitudes as a function of relative period (F). Equations for each model were solved numerically using the Runge–Kutta method with Δt =0.01. (G) Period sensitivity to parameter in negative plus positive feedback model. We prepared 50 basic parameter sets in which v, v_m, k_s, v_d, k_1, k_2, and v_s are one-half to double their standard values (above). For these sets, each parameter was increased by 10% and the corresponding change of the period is shown. Closed circles and thin lines are for the mean and standard deviation of the change.

S3 Fig. Period as a function of temperature when transcription or translation is inhibited 20%. Other rate parameters and activation energies are fixed as in Fig 3. Based on reports that circadian rhythms were maintained even when transcription or translation was strongly suppressed [13,48], period and amplitude are calculated as a function of temperature when overall transcription rate is inhibited by 20% (*k* and *v* in Equations (1) are multiplied by 0.8). Temperature compensation still occurs over a wide range of temperature with partial transcription inhibition (A) and without further parameter tuning. Temperature compensation is also maintained with partial translation inhibition (B) (*s* in Equations (1b) is multiplied by 0.8). C: Period is depicted as a function of relative transcription rate (*b*). In our simple model of Equation (1) and (2), the right hand side of the first term of Equation (1) is multiplied by the rate, *b* for calculation. Other parameters are fixed as in Fig 3 with *T*=308. D: Period is depicted as

a function of relative transcription rate (v'). In the model of Equation (1) and (2), the right hand side of the first term of Equation (2) is multiplied by the rate v'. Other parameters are also fixed as in Figure 3 with T=308. Period is stable over a wide range of translation rates, consistent with Nakajima et al. (2015) who reported that period is relatively insensitive to the addition of a translation inhibitor (cycloheximide) [48].

S4 Fig. Amplitude adjustment for temperature compensation in sequestration model. Distribution of periods when reaction rates are increased for the sequestration model (A). We constructed 2000 parameter sets in which parameters k_D, α_M, β_M, α_C, β_C, α_R, and β_R were increased randomly 1.5- to 2.5-fold above the original parameter set [56]. Sustained oscillations were obtained for 1893 of 2000 sets. We plotted the distribution of both the amplitude of oscillatory mRNA (M, pink) and the geometric mean (green) of the relative amplitudes of M, C, and R are plotted as a function of relative period as reaction rates are increased (B). Values of the original parameter set are: k_D=1e-05, A=0.0659, $\alpha_M = \beta_M = \alpha_C = \beta_C = \alpha_R = \beta_R = 1$.
