# Supplementary figures and images for: Temperature–amplitude coupling for stable biological rhythms at different temperatures
Source: PLoS Comput Biol. 2017 Jun 8;13(6):e1005501. doi: 10.1371/journal.pcbi.1005501 (PMC5464531; doi:10.1371/journal.pcbi.1005501)

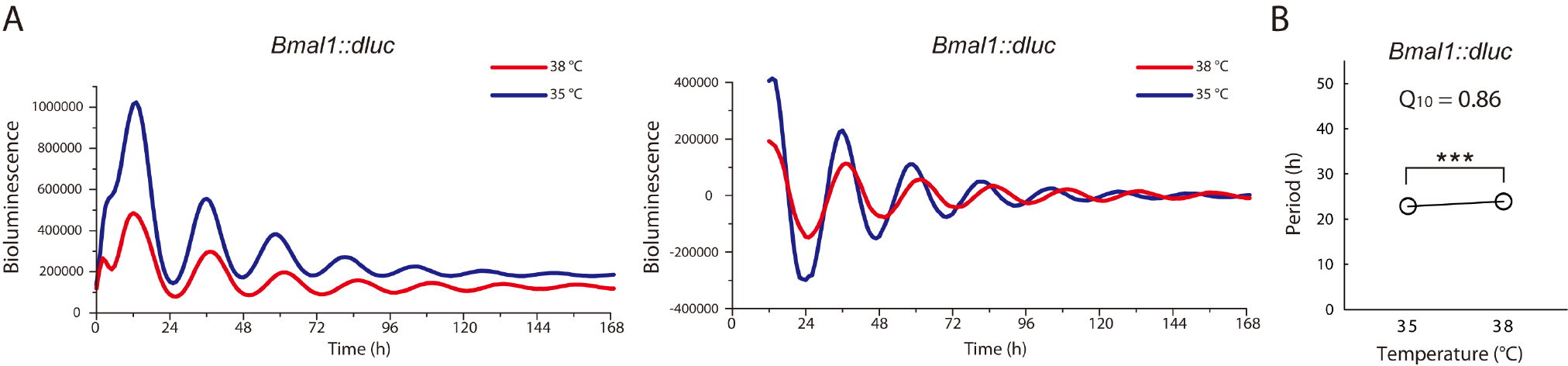

Supplement: S1 Fig — (TIF) [file pcbi.1005501.s002.tif]

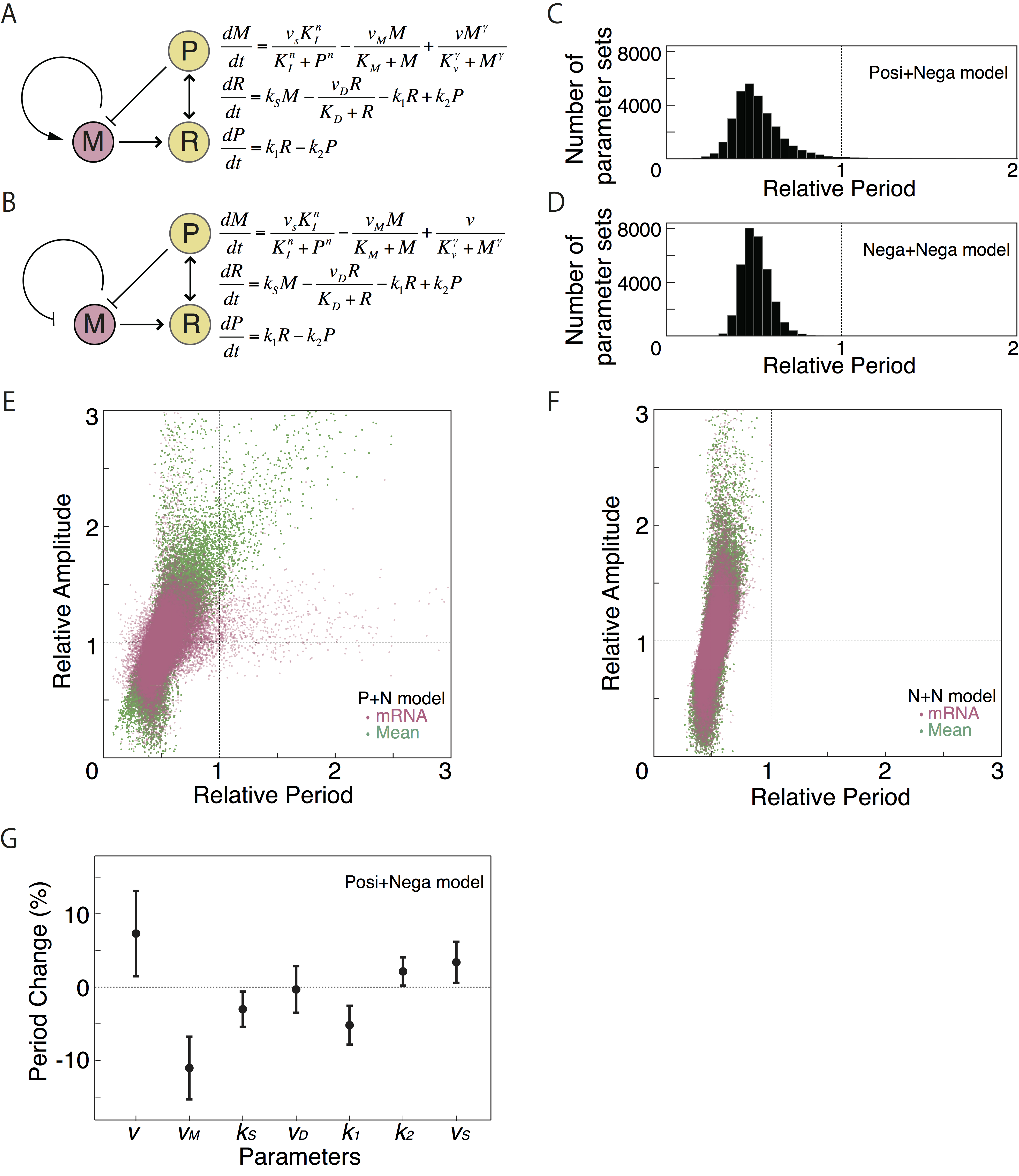

Supplement: S2 Fig — (TIFF) [file pcbi.1005501.s003.tiff]
